# Video connecting families and social robots: from ideas to practices putting technology to work

**DOI:** 10.1007/s10209-022-00901-y

**Published:** 2022-07-30

**Authors:** Isabel Schwaninger, Felix Carros, Astrid Weiss, Volker Wulf, Geraldine Fitzpatrick

**Affiliations:** 1 TU Wien, Argentinierstraße 8, 1040 Vienna, Austria; 2 Universität Siegen, 57068 Siegen, Germany

**Keywords:** Communication technology, Care work, COVID-19 pandemic, Digital literacy, Diary study, Robots, Configuration work, Values, Work roles

## Abstract

Technology use is a socially embedded process, especially when it comes to older adults and care. However, the restrictions associated with the COVID-19 pandemic have limited social contact to protect vulnerable groups in care homes, and even if technology use has increased in other areas, there is little known about the potential uptake of communication technology and changes in social interaction in the care context during a lasting crisis. This paper explores changes in communication technology use triggered by the pandemic at two care homes, using a qualitative diary study, online interviews and observations, and in-situ interviews within the care home with residents and workers. Our findings point to increasing use of tablets and video conference software triggered by COVID-related experiences, with implications for living and working in care homes. We also characterise the isolation experience of the residents, the workers’ concerns about the residents and changes in social interaction. We observed new areas of technology usage, associated changing work practices, technical affinity issues and context-specific attitudes towards future technologies. While the pandemic has triggered the use of communication technology in care homes on a small scale, this has also caused increasing workload and in particular articulation work, which requires support structures and the re-definition of work roles.

## Introduction and background

Technology use is a socially embedded process. When it comes to older adults and care contexts, practices of technology usage are both social and collaborative [35], and are often embedded in care networks [39]. Informal and formal care [28, 35, 48], inter-generational relationships [29] and help networks [11] are important for the use and uptake of collaborative (care) systems. The appropriation and adoption of technology further involves (mainly hidden) configuration work [2, 32, 46].

The care context in central Europe has been however under severe restrictions since the outbreak of the COVID-19 pandemic in March 2020, which caused an increase in physical distancing on the one hand and higher dependency on digital tools on the other. Older adults and other high-risk groups especially have been protected^1^. As a consequence, care homes were closed, visits were prohibited, and resident mobility was restricted. This resulted in a loss of family and intergenerational face-to-face contact [29], and for some even a lack of contact with fellow residents and personnel.

While digital tools may provide an opportunity to promote communication [16] and connection [36] and even to reduce isolation/loneliness [13], residents in care homes do not always see digital tools as an option for stimulation [16] on a physical, cognitive or emotional level. Despite an increased overall tendency to conduct social and economic activities [8, 29] and even research virtually [30], there are yet unequal opportunities to mitigate the risk of social isolation [36], and digital accessibility issues are known from previous COVID research [25]. This engenders the risk of double exclusion in a society that relies heavily on communication technology (CT), namely from physical contact and from digital participation [42]. Further, while COVID-related restrictions have been ordered top-down by governments, earlier studies have shown how healthy ageing is strongly perceived as an active achievement that includes the management of lifestyles, health and illness, and the active balancing of social life and financial and material circumstances [43]. Economic resources and digital skills are crucial factors to reduce risks associated with the pandemic [36], i.e. for social, cognitive and physical stimulation. Both residents and people working in the care sector however often lack the required digital skills [16]; while this problem is not new [10], it has become more pressing when physical social contact is no longer an option.

Special circumstances like crisis situations may reveal societal norms and understandings in a very concentrated way. Further, restrictions may trigger developments and change habits to cope with special circumstances, also depending on the ideas that have been developed and ‘lying around’ [15]. Assistive technology has been developed and discussed to support older adults since many years [20] (e.g. including safety systems, security, monitoring, communication, and entertainment systems, and home automation [20, 49]). The use of technology for communication in particular can be a response to crisis situations [18], and research suggests its potential as a response to isolation in the care context that has occurred since the pandemic [16]. However, there is still little known about the actual use of CT at places like care homes during a lasting crisis. We aim to get a better understanding of how the experience of the pandemic has affected attitudes and use of CT, and associated processes of social interaction at these places.

The overview of this article is as follows. In the next section, we present a description of our methodology, including our mixed-method study and the chronological accounts of the data collection in relation to our research process. Subsequently, we present our findings on changing technology usage associated with experiences of the pandemic, including experienced isolation and changing work practices associated with increasing CT usage, and mixed attitudes towards future technology. We follow with a reflection on solutions to old problems exacerbated by COVID and new issues, along with a discussion on readiness for engagement with new technology, and lessons learned from shifting crisis situations. While the pandemic has triggered the use of CT in the care context to some extent, this also requires solutions for new problems associated with increasing workload which we propose to be tackled in future work.

## Methodology

### Chronological account and context of the study

At the time of the outbreak of the COVID–19 pandemic, we were preparing a study that involved social robots at care homes. However, it quickly became clear that the pandemic had also affected the care context and especially the social circumstances there. We therefore decided to conduct a study on the experiences of the pandemic and its effects on technology usage and changing life and work practices. While no robots were present at this time, we found that other technology was lying around (to be described later in this section), and we were interested in investigating technology usage and attitudes towards future technologies, such as social robots, as a response to this crisis.

**Fig. 1:**
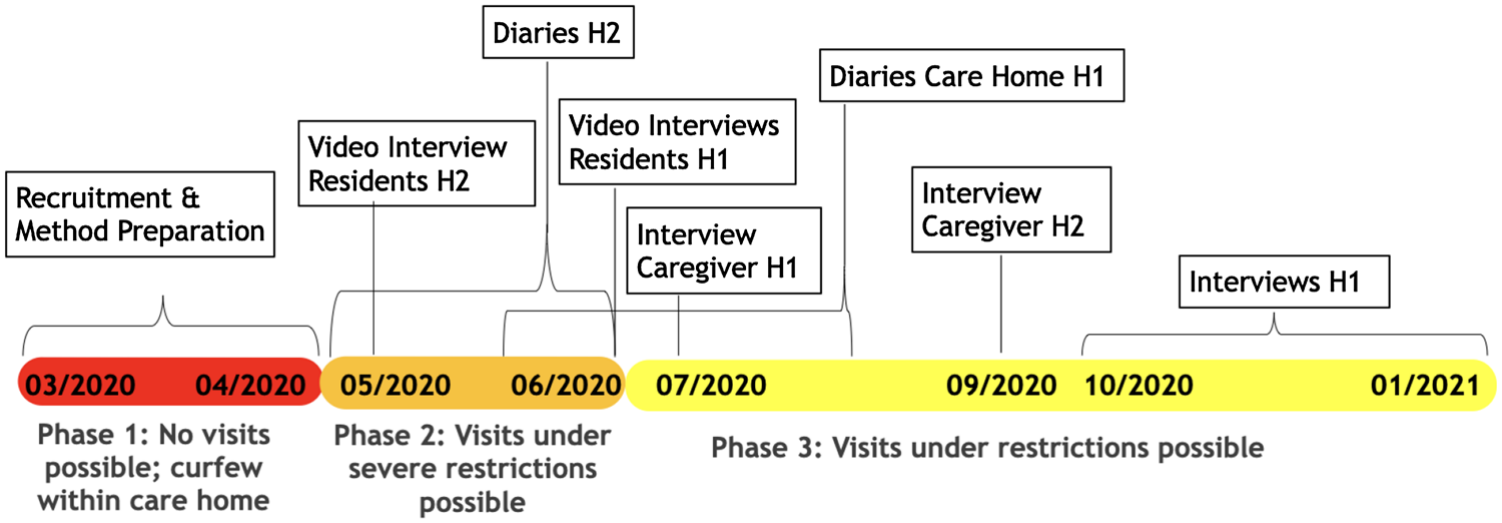
A chronological account of the restrictions and data collection phases

We collected our data in the two care homes at different time phases (see Fig. 1). Phase 1 was the strictest phase concerning the restrictions in Western Europe where our study took place, and it included most of the first lockdown (March–May 2020). In this phase, the care homes did not let other people enter, which also resulted in families being torn apart. We used this phase for method preparation and recruitment. Restrictions to receiving visitors were lifted rather quickly after the first lockdown and they were not reinstated with the same strictness later. In Phase 2 (May–June 2020), visits were allowed under severe restrictions, including window visits^2^, or container visits^3^. A limited number of visitors per day were allowed, enhanced hygiene rules like wearing masks, and rapid COVID tests were in place. In Phase 3 (July 2020–Jan. 2021), visits were possible under lighter restrictions. We collected different types of data in Phase 2 and Phase 3 in the two care homes.

### Research setting and participants

The two care homes that participated in the study are situated in a German-speaking country in Europe. The care homes are anonomyzed in this paper and they are referred to as H1 and H2. H2 is within a big city, while H1 is situated in a medium sized city. H2 is a long-term partner of the university of one of the co-authors and had a regular exchange with the researchers until the pandemic. Over a period of the last four years, the care home worked together and experimented with different digital tools like tablets, smart assistants and social robots. H1 has been also cooperating with the university on additional topics, like education.

Both care homes have religious roots and belong to catholic institutions. They are both connected to multiple other organisations within their buildings (H2: Kindergarten, Residential Assisted Living; H1: Monastery, Administration of Organisation). H2 is approximately twice the size of H1 in terms of permanent residents (119:49). H1 is connected to a monastery with nuns living within the care home.

**Table 1:** The participants’ names as referred to in this paper, their age, gender, care home facility (pseudonymised) and role in the respective care homes

Name	Age	Gen.	Facility	Role
R1	55	m	H2	Resident
R2	86	f	H2	Resident
R3	85	f	H2	Resident
R4	85	m	H2	Resident
R5	84	f	H1	Resident
R6	60	f	H1	Resident
R7	86	m	H1	Resident
R8	60	m	H1	Resident
R9	93	f	H1	Resident
C1	62	m	H2	Social Service
C2	54	f	H1	Management Social Service
C3	19	f	H1	Social Service Intern
C4	36	f	H1	Social Service
C5	52	m	H1	Management Care Home
C6	38	f	H1	Management Care Workers
C7	45	f	H1	Social Service
C8	74	f	H1	Social Service
C9	57	f	H1	Social Service

In the study, we had a total of 18 participants: 9 residents and 9 care workers. The residents were all cognitively able. Before conducting the study, we acquired ethical approval from the university. The residents were invited by the care workers, and their participation was based on their willingness and ability to participate. The residents were between 55 and 93 years old, 4 of them male and 5 female. An overview of the participants is presented in Table 1.

### Data collection and analysis

We conducted a diary study and online interviews with 18 participants (see Table 1 and Fig. 1 for a timeline of methodology) in the two care homes^4^. We collected data from 9 residents by conducting a diary study and interviews (phone or video interviews), and from 9 care workers by conducting face to face interviews.

We created paper-based diaries for residents to fill out for four weeks per participant, with one entry per day. The diaries included open questions and playful activities (such as story completion and postcards (see e.g. Fig. 4) to be sent to researchers and a robot) to gather insights on the role of technology in day-to-day activities, on the experience of social contact and the pandemic, and on perspectives on the next generation of CT (i.e. robots), as we were interested in perspectives on the role of (both current and future) technologies triggered by the pandemic. The diaries were filled out by nine residents at two care homes (i.e. four and five, where the imbalance of numbers has been a result of the voluntary participation). To complement the long-term diary study, the participants who volunteered to fill out diaries were invited to participate in remote interviews with us using a chosen communication medium (e.g. telephone or video telephone using a videoconferencing software like Skype). Four interviews were conducted with residents of H2 via telephone, and five interviews were conducted with residents of H1 via video telephone, with the assistance of a care worker. To gather insights about care workers’ perspectives, we also conducted interviews with nine people working with residents, one from H2 and eight from H1 (based on the workers willingness to participate).

We recorded the interviews using recording software for audio/video. We transcribed the audios and videos and we coded them along with the diaries. The data was analysed with a reflexive thematic analysis approach [4] with the MaxQDA software by inductively clustering different topics. We generated, reviewed and defined themes to interpret results that we present below.

We collected data out of interest in the effects on the experiences of the pandemic on technology use, and therefore did not actively change the environment with research by asking participants to use technology (this was also not possible because of the restrictions, at least in the beginning of our study). However, both institutions had access to technical devices, even if in slightly different ways: Within the 6-years cooperation with the university of one of the co-authors, H2 had received around ten tablets and the residents were trained to use these devices by the university until the pandemic started. H1 on the other hand was not a long-term partner, however they received tablets from a national telecommunication company in Phase 1 and they did not receive any training.

## Findings

Across our data, we identified effects of the pandemic on the care homes, new technology use, and attitudes towards future technology as reported by care workers and residents. In the following, we present findings on experienced isolation, workers’ concerns about residents, and changes in social interaction. This is followed by technology use at care homes, including the use for social interaction and physical activation, associated changing work practices, technical affinity issues, and subsequently, attitudes towards future technology.

### Effects of the pandemic on the care homes

#### Experienced isolation

**Fig. 2:**
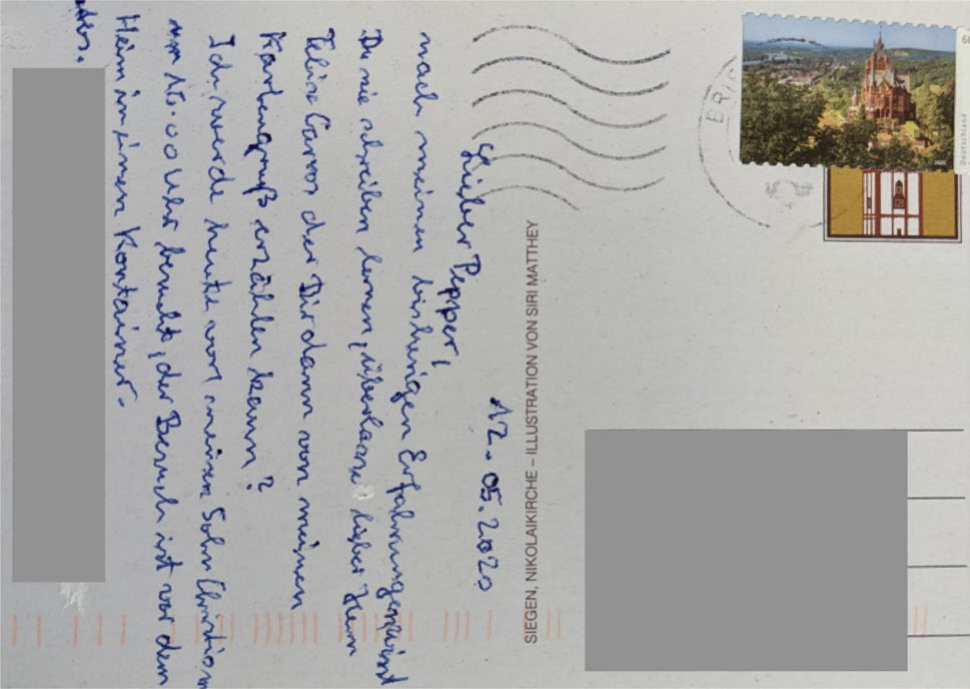
A postcard a resident has written to the researchers describing the visits in containers: *“[...] I will be visited by my Son Christian today at 4 p.m., the visit will be in front of the home in a container. [...]”*

Especially in the first phase, visits were forbidden (“*visits = prohibited!*” (R2)), where later, containers were installed in front of care homes dedicated to visits, as shown in Fig. 2). The restricted visits was difficult for the residents: “*Of course, the [...] people first had to get used to the fact that their people - their relatives - were no longer allowed in the house. That was, I would think, the biggest setback.”* (C2).

Besides the restrictions on receiving external visitors, the isolation of residents was also enforced through restrictions within the care homes. Group activities were reduced: *“Yes, our sports activities and bingo and what we all do. That also has to be cancelled and we can only do it with so much distance you just sit with two men [...] so that we don’t get too close.”* (C1). Furthermore, it became normal that everybody had to wear a face mask within the care homes at all times to protect others from possible infections. These hygiene rules were intended to slow down a possible infection but also resulted in limiting the residents in understanding other people - since their sense of hearing is quite often limited, they depended on being able to use several senses to compensate: *“They had to keep the minimum distance, they had to keep the mask and I would say, the old person can’t hear so well, he can’t see so well. And he depends on it, to see the mouth move and also that you can get close to speak loudly and so on, and all that was not possible. That made some residents feel insecure, rather than comfortable, because you couldn’t reach the other person, so to speak.”* (C2).

The rules also had the consequence that the residents did not receive physical contact when it was not necessary. Before COVID-19, it was normal that a care worker would give them physical contact, like patting the shoulder. Also, visitors normally provided physical contact via hugging or holding hands. All of this was restricted and took away an embodied sensual way of communicating without words.

As the residents are quite a heterogeneous group, not all of them experienced isolation in the same way and they had different perspectives on loneliness. One resident reported that: *“sometimes I need the loneliness”* (R2), suggesting that loneliness has positive sides for her. However, this view was not shared by all residents, as another person told us: *“I want my kids to be allowed to visit me again”* (R3).

#### Workers’ concerns about the residents

Care workers reported safety concerns regarding the residents. On top of the usual health-related risks (e.g. “*the usual influenza will come additionally in November [...], then we are likely to have our noro-virus in the care home [...], as every winter*” (C1)), workers were concerned about not being tested for COVID-19 in Phase 2 (which changed later): “*great that we are not being tested here. Everyone could be a spreader even without symptoms*” (C1). However, there was also awareness that the residents had gone through several other personal and global crises in their lives, where the pandemic may not be perceived as difficult by some of the residents. One resident compared the experience to that of being a prisoner: “*I am 94 now. Four years of war captivity in Siberia, I survived it all. Now I am locked in like a prisoner. I need to die anyway, I want to be able to move around freely during the last months that I have. Within the remaining months I have left, I expect to be able to move freely.” (C1)*.

The care workers recognised that increasing dependency of people as they got older challenged their sense of self-confidence, and the workers would do what they could to try to promote self-confidence: “*in my view, this is the most important, because these people that are here, they all have been like us, [...] they were able to do everything like we do and now all they can do is to experience deficiency [...]. I think the most important for them is that they are able to do it on their own, completely and especially to understand on their own. This will promote their self-confidence and a sense of mastery.*” (C2).

#### Changes in social interaction and communication

Both residents and care workers reported changes in social interaction and communication because of the pandemic. In the care homes, there were fewer group activities happening, and in the beginning of the outbreak they had to eat their food alone in their room (“*lunch is being brought in our room, you know, we don’t go into the big dining area downstairs, and in the morning, a plate with breakfast is put here and in the evening as well*” (R1)). This resulted in more monotonous daily routines, and residents wanted to have more activities and see people (“*of course, then there would be more variety*” (R5)). Residents also made efforts to keep physical distance (“*of course I avoid all the people*” (R2)).

The interaction between residents and care workers changed, where the face masks had an effect on communication. For residents who were depending on facial expressions a lot, the workers “*lose their personality, when wearing this thing, in my view, some of the personality is getting lost [...][my fear is that] everything is getting more mechanical, [...]one is nothing but a number*” (R4). Because of the restrictions, there was also a decrease in face to face interaction with people from outside the care home. Residents were however missing their family members (“*I wish that my children are able to come and visit me again*” (R1)).

### Use of communication technology

#### Using communication technology for social interaction and physical activation

In response to the restrictions and associated experiences, off-the-shelf technology usage was initiated by care workers at both care homes. These were also used because of fewer visits and to increase safety.

To communicate with people from outside the care homes, both institutions were using video telephone from different corporate technology companies (“*we have used WhatsApp a lot, a lot*” (C1), “*a friend usually comes regularly to visit me, now at times of COVID-19 [we have been using] Skype*” (R1)). However, a care worker also stated that video conferencing platforms were used only by approximately 10% of the residents during the pandemic, compared to 0–1% before the pandemic: “*Those who have been using it appreciated it [i.e. video telephone]. Among over 100 people however, if only ten people were using it, this is not a very good quota, because [compared to ]feeling closeness for real, [...], sitting next to a person, [...]*” (C1). However, among the people who were motivated to use communication software, a care worker said: ”*We really took our time for every resident who wanted to be able to use it on their own. Skype is always a bit of an obstacle, but WhatsApp, everyone has it, so we showed it to everyone, even the relatives, how they could use it, so the relatives can contact the residents on their own or the other way around*” (C2).

Video telephone was appreciated a lot, as care workers tried to “*realize everything as good as possible, to take into account every opportunity and guidelines*” (C2). A care home reported how they got support from a national telecommunication provider with tablets and apps such as Skype or WhatApp which made this even easier (C2), and according to the care home, the use of video “*has worked out very well*” (C2). The use of video telephone was also perceived useful for the people who were able to use it. As visits became possible with special measurements, if they “*could compare between Skype and original visits, they preferred Skype, because they said: ‘What should I sit here, where I can’t get close to them and so on, then this is better, I can also hear better etc. when doing it over Skype*!” (C2).

Video conferencing tools were also used by care workers to conduct exercises with residents (“*we continued with our program - movement with music. And we had rehabilitation sport twice a week, and we did the same for movements with music*” (C2)). However, this required a lot of space, “*we had the big advantage that we are very spacious here and we could separate people from each other*” (C2). Workers made use of the videoconference tools with screens: “*with bad image quality and bad audio quality, we used the TV in the cafeteria and in the rooms to transfer and show our sports program*” (C2). People were able to gather and still keep the minimum distance from each other. This way, “*they could still see their usual caregiver on TV’*’ (C2), and another caregiver could still come by to the people and support them physically.

However, the predominant use of tablets for communication required assistance by care workers or relatives. The lack of visits had an effect: “*It [video telephoning] is installed on my laptop. When my son comes, he will help me*” (R2). The help of relatives was missing, which required care workers to assist. With the use of tablets, on the other hand, residents also had more “*access to media*” (C2) to access online information. While residents had been less open to use technology for this purpose before, “*where nothing else was possible, they might have accessed it and say: ‘Okay, now I’ll give it a try’. This was actually very nice.*” (C2).

#### Changing work practices

The use of technology at the workplace entailed changing work practices, with both positive experiences among workers and challenges, and issues related to digital literacy, accessibility and additional workload.

Digital literacy was an issue in general. While care workers needed to assist residents in using communication software, they were also in need of guidance: “*This is currently difficult because of Corona, [however] if there were seminars, also for the residents or small videos with explanations...*” (C3). However, following the engagement with videoconference software to enable residents to communicate with people from outside the care homes, the workers started to use video telephone as a new habit in their private lives: “*This [video] telephoning has become a habit at some point. We also used Skype, everyone who had the opportunity to set it up on their phone*” (C2).

The use of CT also increased the workload. Workers assisted residents with setting up conference software: “*We enabled everyone who had the option to have Skype - with their phone. We guided everyone [...] if they weren’t familiar with it yet*” (C2). Because “*most residents are not able to see very well, so with this, let’s say, video conference with the outside as one could say, they do need a little help*” (C2). In addition, there was also considerable work involved in setting up appointments for the calls: “*people [i.e. family members] called at least a day before Skype calls should happen*” (C2), and “*some [residents] even booked appointments for the entire week*” (C2) to speak to their relatives or friends. During the virtual conversations with relatives, care workers also reported that they were often directly involved e.g. to support the communication and give short updates to relatives (e.g. regarding the status of the resident).

This increase in workload came on top of an already high general workload, exacerbated by restrictions of other people visiting the residents who would normally help. On the other hand, the care workers also found it rewarding to use of technology. They experienced learning they were able to support the residents’ needs (“*This was actually great*” (C2)).

#### Technical affinity of care workers

**Fig. 3:**
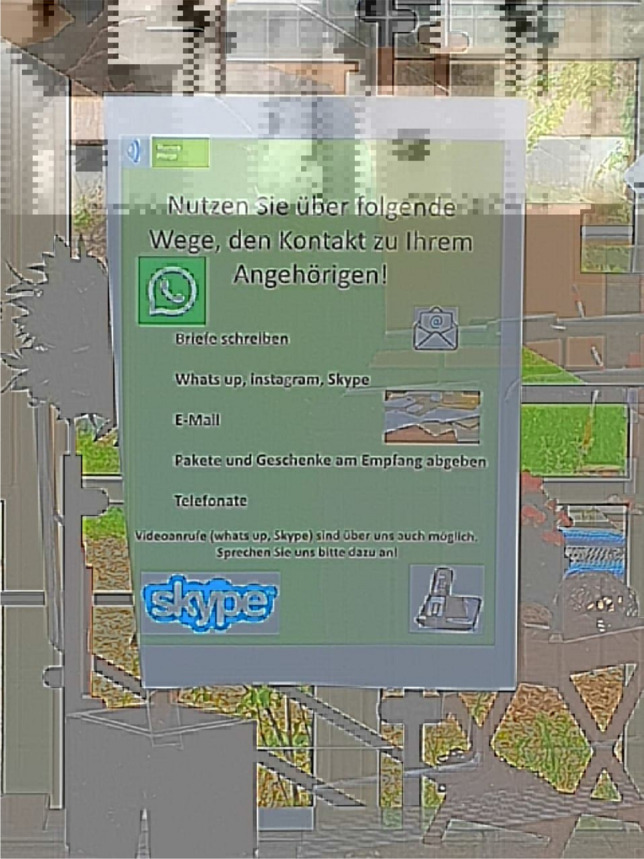
An advertisement at H2 to communicate with relatives via CTs like Skype and WhatsApp

As care workers were forced to use CT at their work and because they have used video telephone frequently (“*we have used WhatsApp a lot, a lot*” (C1), see also Fig. 3), their technical affinity appeared to increase. While care workers had not expected to need technology for their work before the pandemic (C2), “*once that nothing else was possible, they might have have accessed the technology, ‘okay, now I’ll give it a try’. This was actually nice. Also the fact that we have to engage with it – what is possible, what is good for everyone’*’ (C2).

One care worker expected the new habits to remain at the care homes after the pandemic. Videotelephone “*[...] will not be gone only because of opening steps, instead, this [i.e. videotelephoning] is an additional option now [...] Of course, Skype will play a role in the future, the same goes with WhatsApp calls with video. This will not just disappear because of the opening, but it is becoming an additional option*” (C2).

However, while they accepted that the use of technology would persist, they were also aware of their skills gap. The generally low technical affinity of people working at care institutions (i.e. care workers and people in management positions) was mentioned (C1). Technology usage would mean additional workload. A care worker however stated that there were too little offers from the government to promote digital literacy (C1).

### Attitudes towards future technology

**Fig. 4:**
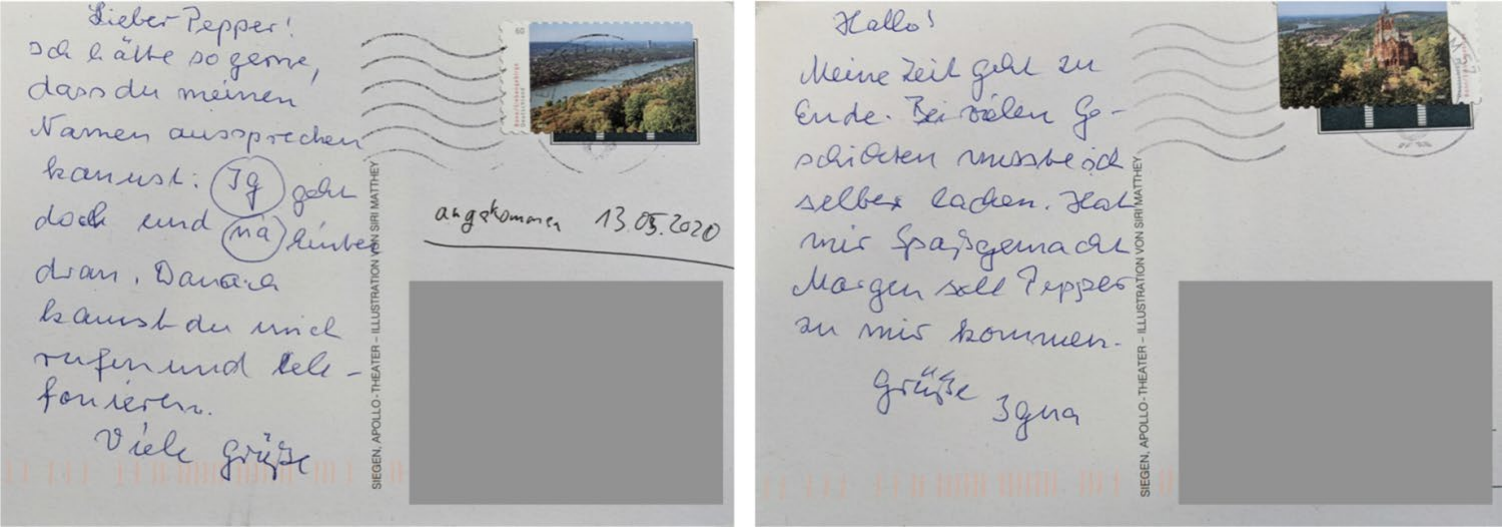
Two postcards a resident sent to the Pepper robot

As we also asked questions about attitudes towards robots (i.e. our intentional focus before the pandemic had started), we found mixed attitudes from residents. Some residents were sceptical about the potential for robots: *“Modern technology can open new perspectives but can never replace human affection!”* (R4). The resident sees technology as not smart: *“Computers don’t think by themselves. Everything has to be inputted beforehand.”* (R4). This opinion is not shared by everyone though as can be seen in a comment that was made about a robot from previous experiences at H2: *“Yes, I like him. He is not too tall, he doesn’t frighten me and I want to have him with me”* (R3). A resident who had experiences with Pepper wrote postcards to the robot, saying *“I would love that you can pronounce my name [...]”*, and *“[...] tomorrow Pepper should come and visit me”* (see Fig. 4). One resident who had previous experiences with Pepper even reported that his family was interested in the robot: *“My kids want to get to know Pepper.”* (R2).

A care worker on the other hand expected a social robot to be problematic for residents because of their very feeling of deficiency: *“They are likely to build up a relationship right away [...] ‘oh he is cute’, ‘he is so pretty’ [...] I believe they wish for too much, even if unconsciously. And if Pepper [i.e. the robot] does not react to what I would be able to provide, a person who is used to bear a lot of deficit may feel deficient again. [...][T]hey will think immediately, ‘is it my fault’, ‘why does he [Pepper] not understand me’...*” (C2).

The residents and care workers had many ideas regarding the functionalities of a robot and the tasks it could do for them. One care worker saw it as something that would be helpful to give orientation to people with dementia: *“For the elderly, demented it could be something to give them orientation. [...] Maybe saying things like: ‘At noon we have this and this as lunch’ or ’This morning at 10:30 the Bingo game will happen’* (C2). The residents saw several tasks that a robot could do like cleaning or bringing things from one place to another. A resident who had previous experience with Pepper had a specific idea on how he would cooperate with a robot: *“I am taller than him, I will work on the things that have to be done on the wall and he can help me on the ground.”* (R3). A resident also thought the robot could measure the temperature of people (R2). One care worker however expected the robot to promote physical contact: *“it is rather perhaps even contact-promoting or also motivates, rather to the participation in groups. And that could be counterproductive, of course, regarding Corona”* (C1).

## Discussion

The aim of our study was to get a better understanding of how the experience of the pandemic has affected attitudes and use of CT in care contexts. When looking at the results, many issues that were described by participants (such as family members living far away, the loss of family members because of death, or low digital literacy) were not entirely new but became apparent in a condensed way. CT and its usage at care homes has been an idea ‘lying around’ [15] to be exploited, however, it took a pandemic to increase the use of these technologies (even if on a small scale) to respond to these old problems. Furthermore, as CT use has evolved, new issues appeared associated with increasing workload and the configurations in which CT has been put to work.

The changes in social interaction through the usage of CT are also reflected in values that we identified across different contexts and time phases: social connectedness and autonomy. In the following, we unpack these values and we make suggestions how to promote them in the future. Subsequently, we discuss readiness to engage with CT, followed by lessons learned from shifting crisis situations, where we pull out implications for living and working in the care context where technology is now expected to stay: the need for the acknowledgement of increasing workload and support structures on several levels, and the re-definition of work roles and processes. Furthermore, we discuss an outlook taking into account the next generation of CTs, followed by methodological remarks and limitations.

### Values in social interaction

#### Social connectedness

The restrictions of the pandemic [51] have further reinforced the notion of care homes being isolated in society. As a group prone to higher risks, older adults living in care homes have been experiencing isolation at times during the pandemic, with fewer visits and increased restrictions in mobility at times that have affected social interaction.

Experiences of loneliness or solitude were mentioned not only in relation to the restrictions. They were also associated with losses of relatives or partners that residents have experienced previously (i.e. before the pandemic) or with relatives living further away (not caused by the pandemic), as also discussed in the literature [12, 13, 41, 44]. Further, experiences of loneliness were positively and negatively connoted, where it is important to take into account that loneliness does not simply mean the absence of people but it has been also described as the absence of context and connectedness (which is not necessarily provided by the presence of people, as one can also feel lonely in groups) [9]. As residents were open to new forms of social interaction and communication that has also involved technology, it is important to aim for connectedness and context in a meaningful way, also taking into account specific experiences of loneliness.

There were accessibility issues with face masks for residents who were dependent on reading facial expressions during face-to-face communication. It is therefore important to design hygiene rules in a way to enable communication and connectedness where possible, especially given the impact these rules had on social interaction and communication.

CT was introduced and used for social interaction. It took a pandemic to discover the potential of these devices in the care context for certain tasks. Care workers also reported positive experiences of using tablets in their work, as they described the use of video conferencing tools to promote a sense of community. Residents who have been able to use video telephony to communicate with relatives appreciated it, where the social potential for digital technology for older adults in general has been already highlighted in previous work [19]. Advantages were even mentioned compared to face-to-face visits if these visits were restricted: people were able to hear/understand better as compared to visits without being able to touch each other or the wearing of masks. However, digital competence is a requirement and digital literacy issues excluded a lot of people (a known barrier for technology use among older adults [14] also in previous COVID research in the care context [16]), or they required the help of care workers to make contact with people outside care homes. We therefore see digital literacy a key requirement to promote social connectedness with digital tools. On the other hand, promoting digital literacy may be also used to establish social connectedness, e.g. by creating social activities around digital literacy promotion.

#### Autonomy

Using CT has the potential to promote residents’ autonomy in daily life (e.g. through access to additional information and / or communication). However, as shown in previous work [22], autonomy is a multi-faceted concept [5] and sometimes one facet can be impaired to ensure another one. While some residents interacted with relatives virtually, these modalities of communication also came with new dependencies due to the additional articulation work [37] (to be further described in Sect. 4.3): Residents required care workers to assist them in setting up and actually being in contact with people from outside care homes, and schedules were made in advance. This articulation work may involve choices to be made for residents to promote their autonomy, for example, in when, how and with who to communicate.

Autonomy is also tied up with mobility and the regulation of the environment with regard to mobility, where restrictions forced people to have fewer social contacts and to move around less freely. Our data was collected in settings and with residents with little mobility (i.e. compared to the rest of the population), and residents also expressed that they were not happy with these sorts of restrictions when they felt they had little time left. Therefore, the restrictions in autonomy (i.e. not being able to move freely) may be perceived in a particular way at older age, especially as people living at care homes may not have the same choice of following or breaking such rules autonomously.

### Readiness to engage with CT

The pandemic has stimulated residents and care workers to deal with the impact of the pandemic by trying out new forms of social interaction and communication, especially with the use of CT like tablets. Care workers also experimented with video conference tools to conduct physical activities and support people. At some point, residents were also open to try out the use of tablets for information retrieval and to communicate with relatives.

However, digital communication practices have varied among older adults even long before the pandemic [3, 23], which is also reflected in our data showing only a small group of residents have actually used these new devices. Here, it would be possible to make these residents internal facilitators for other residents to help them with the use of tablets, as it has been the case in other research (albeit with active older adults not living in care homes) [39]. This could also be used to meet known barriers among older people such as asking for assistance with technology usage [14].

However, readiness to engage with technology that is lying around depended on various factors: Obviously, a crucial factor was the availability of tools lying around. Further, the willingness of care workers to support residents in digital communication was also crucial and required them to actively engage with technology. A lack of digital competence was also mentioned in one care home, tied to a lack of support by the government, social security and in care workers’ (ongoing) education. Care workers needed to adapt their work practice to the new forms of support they were giving to the residents and by doing so changed their work habits from helping family members to meet the residents within the care home to helping the residents and the families to use this CT. Readiness to engage with new technology could be promoted through the availability of devices, training and other support structures, institutional (work) culture, working conditions and the roles of workers. This has implications on relationships and the coordination of care networks, which connects to previous research in the care and healthcare context [17, 31, 50].

### Lessons learned from shifting crisis situations

*Crisis situations can change imaginaries of what is possible to respond to old and new problems.* Digital transformation has been accelerated in various sectors during the pandemic [8, 13, 27, 29], and we see changes also in the care context to some extent. Most notable however, the pandemic experience has changed some imaginations of what is possible or what is valued, where solutions for old problems were found. Many relatives were already living far away from residents, resulting in very few visits from them and very little contact. The problem persisted. While technology had been there and relatives had been sometimes far away, the pandemic experience has triggered the usage of CT.

*Engagement with new CTs and associated care practices requires an ecosystem perspective with interdependencies at least between politics, organizations, workers and residents.* Care workers mentioned political agenda requirements to support the use of technology during work and to include aspects of digital competence into education and ongoing education of care workers. Institutions across care and politics can offer these opportunities and by doing so enhance digital literacy and access to state-of-the art technology. On an organizational level, the willingness of care workers and residents is needed to engage with technology in day-to-day work and life. The care institutions are also the ones who engage in institutional collaborations, for example, with research institutions and private corporations. Such corporations can provide access to participation in development, where participation can provide learning for older adults [26]. The care workers also need a certain level of readiness to engage with new tasks and use technology to fulfill their goals in day-to-day work, provided that they are offered opportunities by care institutions to do so, and that the technology is developed for them to actually support their daily work.

Last but not least, residents need a certain level of digital competence. This connects to previous COVID research which suggests a perspective to understand digital tools as they are being used by residents, not merely as an instrument but also as a learning process that needs professional support, infrastructure and training [16, 34].

*The use of new CTs requires a collaborative perspective as they involved a triune of residents, care workers and family members.* For the most part, digital devices were not used in a dyadic human-human interaction between a resident and a family member, nor has there been an omniscient and omnipotent agent [37] or user, but they have been used in a triple of residents, care workers and family members. The three parties are needed to establish a connection between each other. The family calls the care worker to make an appointment, the care worker then makes an appointment between the resident and the family. Once this is established, the communication still needs additional support. The care worker sets up the video call and quite often stays in the room with the residents to help out and to give an update to the family. This also means that all three parties have to appropriate a new technology and learn about the changing dynamics that come with it, where they need to articulate the distributed (work) activities [37] to interact and communicate in spite of distance. For the resident, it adds a layer of transparency, since in this situation they are present and talks between the family and the care worker about the resident and their general well-being are done with them instead of outside of their room. As shown by Lahtiranta et al. [24], care workers are building a bridge between the people who are not used to the technology and the technology itself. Furthermore, they are mediating the bridge to the family.

*The use of CT adds to the workload which requires recognition, support structures and new work roles.* The workload of care workers was increased during the pandemic also because of the three party arrangement. While one could argue that fewer visits may have freed up some time for the workers, health and care sectors may also rely heavily on adult family members’ support [29]. Also, care workers have mediated technology use on top of extra pandemic-related workload (e.g. due to changing hygiene concepts and evolving rules that came with the pandemic), which is related to the (mostly hidden) configuration work [2]. Care workers however saw a value in supporting residents to virtually communicate with family members and to use technology for sports and entertainment (even though one could argue that they were running the risk of extra workload in the future). This may have an impact on the organization of work and we see a potential for new work roles, as it is not clear yet who is going to formally take the work that technology brings in. Therefore, roles of care workers need to be re-defined, which may also involve changing job descriptions and specific time allocated. There is also a need for increasing staff and support structures to change work processes (which may also involve union involvement). In order to be sustainable, the work that technology brings in needs recognition and support. Furthermore, even if some older adults may be able to use technology independently after a while (as shown in previous case studies [45]), this may not be always the case given various barriers [14].

### Outlook: implications for the next generation of CTs

Before the pandemic started, we were in the middle of planning a study with a social robot to be deployed at the care homes. However, at the time of the outbreak in Europe, this has not been possible. It was the idea of engaging with off-the-shelf CT ‘lying around’ [15] that has been available to be picked up, not social robots.

While CT (i.e. tablets) that has been exploited during the pandemic may be much different from a robot, it is still worth discussing an outlook for future technologies and what can be learned from responses to this crisis for the next generation of CTs. This does not mean that the findings are directly transferable, especially as expectations towards robots are very specific and grounded in pre-conceptions [40]. However, it also became very clear in our study that certain aspects were not a matter of technology itself, but a matter of the context of living and working at care homes in how CT has been integrated into work and life.

First of all, readiness to engage with technology needs to be taken into account. Here, political and institutional requirements are important as well as the role of workers and residents that are not necessarily a matter of technology but a matter of the context. While robots are however not “lying around” usually, a limitation is here that robots may require a different set-up and even more training than already discussed in the case of these much simpler devices, i.e. tablets. The moderator role of care workers as part of a triune that we identified in the use of tablets may be even more important to consider for the use of robots (which also connects to previous work stating that the actions of care workers are crucial for facilitating human-robot interaction in certain care processes [6, 7, 21]). While robots are often envisioned in a dyadic human-robot constellation [21, 38], it is important to acknowledge the dependency of residents on care workers and their key role in configuration work. Our study has shown that CT usage can lead to new dependencies which requires future work to explore opportunities for new work roles or additional staff. While it is often discussed that robots offer more *intuitive* ways of interaction [1, 33, 47, 52], the reasons for this additional workload is also grounded in the task itself (rather than only interaction modalities of a technical artefact, which is nevertheless a key design aspect too). The work practices and ways of day-to-day care along with the crucial role of care workers for putting the communication with relatives to work must be certainly acknowledged also when designing and integrating future generations of CTs in this context, which also needs further exploration in future work.

### Methodological remarks and limitations

On a methodological level, we aimed to adapt our research process continuously to the special needs and conditions of this time. This research also involved sensitive or difficult topics such as social isolation, experiences of being alone and related issues. Because methodology can affect people especially when they are being confronted with sensitive questions, we took a lot of time, research and preparation ahead to develop material (i.e. the diaries and the interview questions asked). The feedback has been mostly positive as people were able to reflect when filling out a diary, also dealing with emotions. As some care workers needed to assist people to fill out their diary (adding more work), this may have also facilitated a connection (and this very fact may have affected the data to a certain level). The postcards may have also shown how the residents connected with us and we got a better sense of their experiences.

Nevertheless, we were mostly constrained to use virtual data collection methods. While we explored different methods (e.g. diaries, story completion, postcards, and virtual interviews), there may be more suitable methods to gather insights of the field, such as in-situ interviews or ethnographic methods. The assistance by care workers in filling out diaries may have resulted in biases. Furthermore, we discussed the three party arrangement which involves residents, care workers and family members. We focused on the residents and the care workers as a key part of this arrangement. A limitation of our research is that we have not collected any data from the family members with this party as such, and which we propose to do in future work.

Last but not least, the pandemic came abruptly and affected life and work (not only at care homes), which has caused stress that has persisted. One of many other stress factors is the risk of infecting a highly vulnerable group. A limitation of this study may be therefore that the work practices may not be representative, as care workers may have also suffered from the effects of the pandemic in regard of metal and physical health.

## Conclusion

The restrictions in care homes during the pandemic have caused old problems to become more pressing, such as low digital literacy and distance of residents’ family members. In addition, new problems like experienced isolation, fewer visits and changing interaction and communication practices have been associated directly with the pandemic. The pandemic has triggered increasing technology use to respond to old and new problems. This has also entailed changing work practices of care workers and increasing workload. Because it is likely that technology is there to stay, future work needs to tackle the changing work and communication practices associated with technology usage at care homes. We see implications like the need to re-define work roles and increased staff, the need for support structures to tackle issues of digital literacy and support for ongoing education and support for teaching skills for care workers to support residents’ independence in digital communication. For a more equal access to CT, it is important to provide multi-level support on taking into account different levels of digital literacy among care workers and residents in future work.

## Footnotes

1 On March 11th, Dr Hans Henri P. Kluge, WHO Regional Director for Europe has announced the pandemic: “*COVID-19 impacts the elderly and those with pre-existing health conditions most severely. In a spirit of solidarity, we all have to be ready to contribute our part to protect those people at highest risk. As individuals, practising good hygiene and prevention measures as well as applying measures of social distancing, including avoiding crowded places, continue to be very important.*” [51]

2 i.e. visitors communicated with the residents through a window without entering the care home

3 i.e. containers were installed in front of care homes especially for visits

4 Note, we did not intend for a comparison between the two care homes. However, we had several reasons for inviting two institutions to participate in this study: (1) We were in the middle of a collaboration with the two institutions at the time of the outbreak of the pandemic, (2) we wanted to get a broader view on the topic, (3) the situation and topic was delicate and it required a highly exploratory approach, and (4) only four residents volunteered to participate in our virtual study in H2 who we had invited first. Methodologically, the only difference in approaching the institutions was the slightly different time of data collection (as shown in Fig. 1). Furthermore, as described earlier, we conducted telephone interviews with the residents in H2 and online video interviews with residents in H1 responding to their preferred medium.
